# Stroke patients’ utilisation of extrinsic feedback from computer-based technology in the home: a multiple case study realistic evaluation

**DOI:** 10.1186/1472-6947-14-46

**Published:** 2014-06-05

**Authors:** Jack Parker, Susan Mawson, Gail Mountain, Nasrin Nasr, Huiru Zheng

**Affiliations:** 1 ScHARR, University of Sheffield, Innovation Centre, 217 Portobello, Sheffield S1 4DP, UK; 2 Computer Science Research Institute, University of Ulster, Jordanstown campus, Newtownabbey, Co. Antrim, Northern Ireland BT37 0QB, UK

## Abstract

**Background:**

Evidence indicates that post − stroke rehabilitation improves function, independence and quality of life. A key aspect of rehabilitation is the provision of appropriate information and feedback to the learner.

Advances in information and communications technology (ICT) have allowed for the development of various systems to complement stroke rehabilitation that could be used in the home setting. These systems may increase the provision of rehabilitation a stroke survivor receives and carries out, as well as providing a learning platform that facilitates long-term self-managed rehabilitation and behaviour change. This paper describes the application of an innovative evaluative methodology to explore the utilisation of feedback for post-stroke upper-limb rehabilitation in the home.

**Methods:**

Using the principles of realistic evaluation, this study aimed to test and refine intervention theories by exploring the complex interactions of contexts, mechanisms and outcomes that arise from technology deployment in the home. Methods included focus groups followed by multi-method case studies (n = 5) before, during and after the use of computer-based equipment. Data were analysed in relation to the context-mechanism-outcome hypotheses case by case. This was followed by a synthesis of the findings to answer the question, *‘what works for whom and in what circumstances and respects?’*

**Results:**

Data analysis reveals that to achieve desired outcomes through the use of ICT, key elements of computer feedback, such as accuracy, measurability, rewarding feedback, adaptability, and knowledge of results feedback, are required to trigger the theory-driven mechanisms underpinning the intervention. In addition, the pre-existing context and the personal and environmental contexts, such as previous experience of service delivery, personal goals, trust in the technology, and social circumstances may also enable or constrain the underpinning theory-driven mechanisms.

**Conclusions:**

Findings suggest that the theory-driven mechanisms underpinning the utilisation of feedback from computer-based technology for home-based upper-limb post-stroke rehabilitation are dependent on key elements of computer feedback and the personal and environmental context. The identification of these elements may therefore inform the development of technology; therapy education and the subsequent adoption of technology and a self-management paradigm; long-term self-managed rehabilitation; and importantly, improvements in the physical and psychosocial aspects of recovery.

## Background

Stroke is a global problem and the worldwide incidence of stroke is set to escalate from 15.3 million to 23 million by 2030 [1]. In the UK, strokes are the largest single cause of disability [2] costing the economy of £8.9 billion a year [3].

Evidence indicates that post − stroke rehabilitation improves function, independence and quality of life providing that it is long-term, intense, task-specific, context-specific, goal-orientated, variable, environmentally enriched and crucially, includes feedback on performance [4-9]. However, due to ever increasing service demand combined with financial constraints, needs for stroke rehabilitation cannot be met [10]. Innovative interventions and service models are recognized as being an essential means of delivering the changes that are required to meet the challenges faced by healthcare [11]. Solutions that allow rehabilitation to continue beyond the acute period thereby improving outcomes, place less demand upon services and enables stroke survivors to be less reliant upon services and able to self-manage are essential [9,10,12,13].

Recent technological advances have prompted the development of Robot-Assisted Movement Therapy [14], Virtual Reality Technology [15] and Inertial Tracking Devices to assist with stroke rehabilitation [16-18]. These devices have the potential to provide consistent, detailed, individually adapted feedback in the absence of the therapist [19]. Furthermore, such systems may also have the potential to promote self-managed rehabilitation in the home, over the longer term [20].

However, the drive towards the use of new technologies and the promotion of self-management leads to further questions regarding the reliance a stroke survivor may have upon a therapist for both, motor learning skills [21] and the self-management of the resultant long-term disability [13].

### The Self-Management Supported by Assistive, Rehabilitation and Telecare Technologies (SMART) programme

The EPSRC funded SMART Rehabilitation research programme (http://www.thesmartconsortium.org) [22], began in 2003 to develop and test a prototype telerehabilitation device (The SMART Rehabilitation Technology System) for therapeutically prescribed stroke rehabilitation for the upper limb. The aim was to enable the user to adopt theories and principles underpinning post-stroke rehabilitation and self-management. This included the development and initial testing of the SMART Rehabilitation Technology System (SMART 1) [16,17] and then from 2007, a Personalised Self-Management System for chronic disease (SMART 2) [23,24].

### The SMART rehabilitation technology system

**Figure 1:**
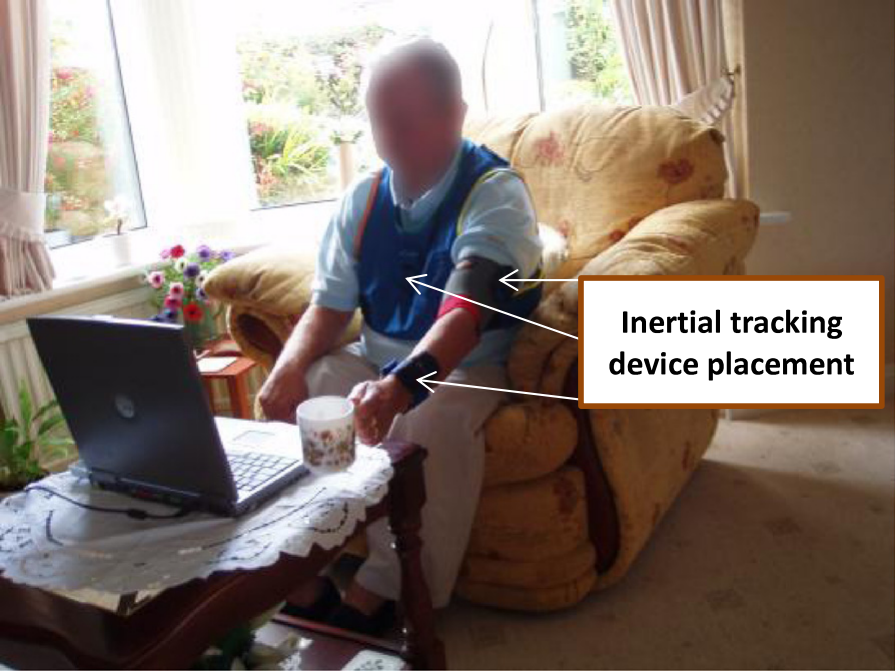
**User participation with The SMART Rehabilitation Technology System in the home-setting (Mountain et al.**[17]**).**

The SMART system incorporates a wireless sensor system that allows for three dimensional (3D) real-time computer feedback whereby the user is able to view the avatar from various planes of view [17]. The system uses inertial tracking devices worn on the upper arm, wrist and chest to record kinematic data from the users when they are undertaking the prescribed exercise [24]. Figure 1 illustrates a previous participant using the system.

**Figure 2:**
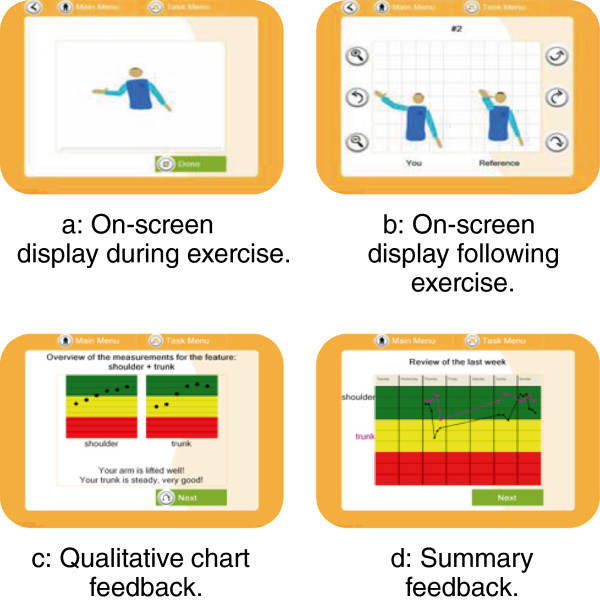
Feedback Screens (a) Concurrent knowledge of performance feedback (b) Terminal knowledge of performance feedback (c) Knowledge of results feedback (d) Summary feedback.

The SMART system monitors and tracks the upper arm rehabilitation movements in real time. It allows motion patterns to be identified, analysed and corrected by both the patient (during and after use), and the therapist (on review) [25]. The program is designed to enable recording and playback using an avatar presentation (Figure 2a, b) and provides qualitative knowledge of performance describing the characteristics of performance (i.e. you lifted your arm higher that time) (Figure 2a and b), and knowledge of results describing the result of a performance (i.e. you scored 88%) (Figure 2c) and summary feedback (feedback provided over a period of time) (Figure 2d) in chart. This feedback allows the user to analyse and record the movements performed in the absence of a therapist [25]. Crucially, in order to encourage self-appraisal the playback facility allows the user to analyse and compare their movement to a reference figure displaying the correct movement pattern (Figure 2b).

Initial findings from technology deployment revealed how a number of usability and contextual issues impacted on the theoretical outcomes underpinning this complex intervention [17]. Further investigation was required to explore how utilising computer feedback for upper limb stroke rehabilitation in the home setting can impact on the underpinning theory-driven mechanisms and theoretical outcomes.

This paper describes the application of an innovative evaluative methodology to explore the utilisation of feedback for post-stroke upper-limb rehabilitation in the home [26]. This includes refining the underpinning theories embedded within the SMART system.

## Methods

**Figure 3:**
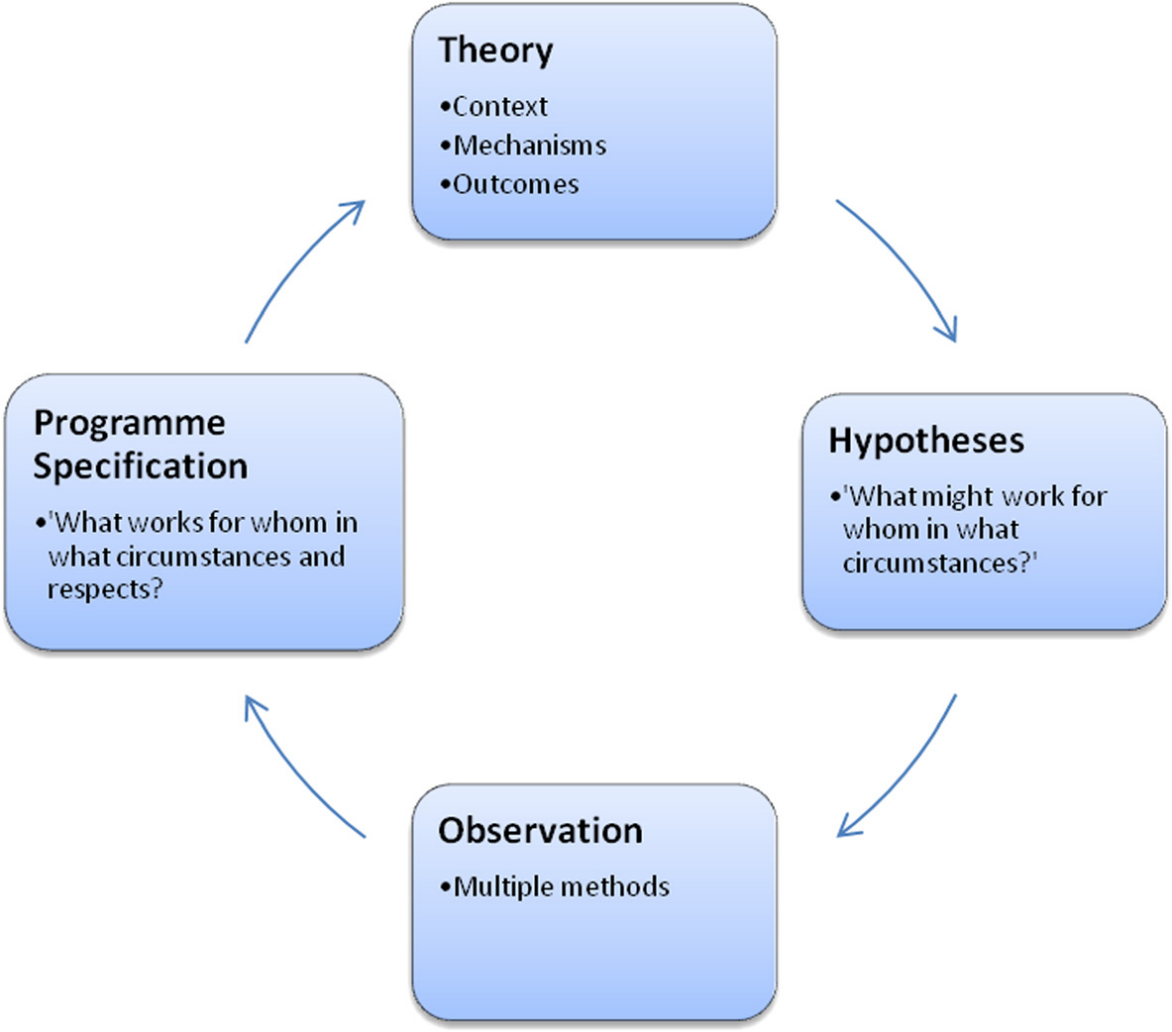
**The RE framework in this research (Pawson and Tilley**[27]**p.58).**

Realistic evaluation (RE) explores how a mechanism may lead to differences in outcome if it is added to a different context: *Context + Mechanism = Outcome*[27]. Figure 3 illustrates the RE process and framework used in this research.

Definitions:

*Context –* The conditions that enable or constrain the mechanisms.

*Mechanism* – What is it about intervention A that produces B?

*Outcome(s)* – The intended or unintended consequences of interventions [27].

Using the principles of RE [28], focus groups with Community Stroke Teams and preliminary testing of the SMART system in participant’s homes were carried out to validate and refine hypothesised context-mechanism-outcome configurations (CMOCs). This was followed by systematically testing the refined CMOCs through multi-method case studies using the SMART system for a prolonged period (up to five weeks). Data were analysed in relation to the hypotheses case by case. This was followed by a synthesis of the findings to answer the question, *‘what works for whom and in what circumstances and respects?’*

### Theoretical framework and outcomes

Technology development took account of the theories underpinning motor re-learning, self-management and behaviour change. To achieve this, the theory-driven mechanisms such as, how receiving feedback from computer-based technology might encourage independent, self-evaluation and self-monitoring of recovery, were included to encourage users to carry out intense, repetitive, self-directed rehabilitation based on goal setting and actively problem-solving [29-31]. To achieve optimum outcomes, these mechanisms had to be able to support this behaviour over time.

### The generation of context-mechanism-outcome configurations (CMOC’s)

**Figure 4:**
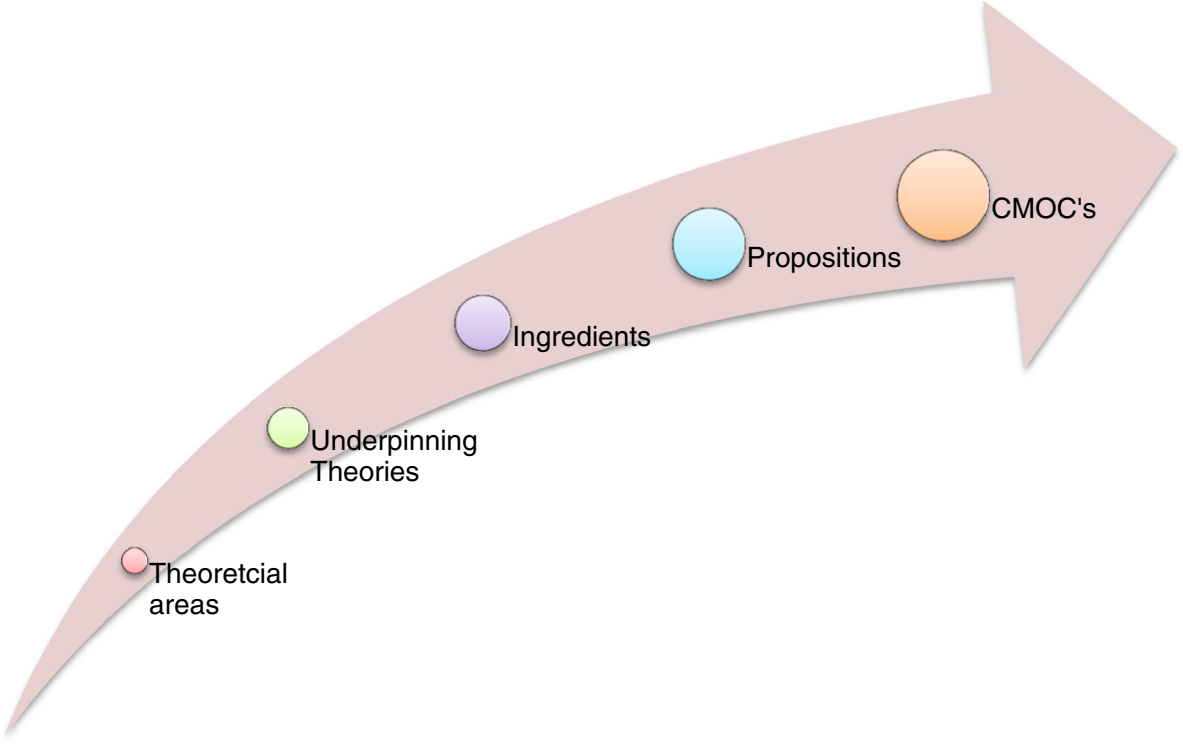
The generation of CMOC’s.

The process of generating CMOC’s involves exploring the theoretical constructs underpinning the intervention. These lead to propositions which are then set out as CMOC’s. Figure 4 illustrates the process of moving from theory to the generation of CMOC’s (Figure 4).

The theoretical ‘ingredients’ that are drawn from the theories; how the feedback from the SMART system incorporates these ingredients are set out in Table 1. The resultant propositions are then set as mechanisms to describe the *process* of how users of the SMART system act upon the intervention stratagem to make them work. These are then set as CMOC’s. The final part of developing the CMOC’s involves describing possible outcomes. An example of two of the CMOCs proposed is presented in Table 2.

**Table 1:** The incorporation of theory-driven ingredients within the SMART system

**Theory Topic**	**Theories**	**Ingredients**	**How the feedback from the SMART system incorporates these**
Theory Topic 1: The theoretical approach to post-stroke rehabilitation incorporated within the SMART system.	Underpinning Theories: Neuroplasticity; Motor-learning	Independent practice	Used in the absence of a therapist.
		Intensity	Increased rehabilitation activity.
		Problem solving	Self-monitoring, self-interpretation, overcoming problems encountered in the absence of a therapist.
		Goal setting	Choosing which exercises to perform.
		Specificity	Matched movement patterns.
		Repetition	Increased rehabilitation activity.
		Salience	Relevant (meaningful) feedback.
		Motor learning	The SMART system provides an opportunity to learn implicitly and explicitly through trial and error and explicit feedback.
Theory Topic 2: Feedback in Post-Stroke Rehabilitation.	Underpinning Theories: Motivation; Operant Conditioning; Motor-learning	Feedback content	The SMART system provides KP, KR, verbal and visual feedback. It also provides prescriptive feedback (in part).
		Feedback schedule	The SMART system provides feedback concurrently, terminally, after each performance and in summary.
		Rewards	The SMART system provides the user with the rewards of good performance through a red, amber, green chart and through scores (depending on exercise).
Theoretical Outcome: Behaviour change and Self-management.	Underpinning Theories: Self-regulation, Social cognition, and goal-setting theories; Self-efficacy.	Goal-setting	The user is able to set specific, measurable, realistic, and time specified goals (targets) to achieve that are confirmed by the computer feedback.
		Action planning	The user can choose when to use the system and how many sets/repetitions they do.
		Self-monitoring	The user is able to monitor performance(s) independently.
		Reinforcement	The SMART system provides the user with positive feedback (depending on performance). Others are able to observe results.
		Self-management	The SMART system provides the user with an opportunity to problem-solve, make decisions, utilise resources, collaborate with others, and take action depending on their interpretation of the feedback provided.
		Self-efficacy	The SMART system provides the user with an opportunity to evaluate achievement(s), observe demonstrations (the avatar), interpret performance(s) and changes in physical and emotional feelings as a result of usage, and receive feedback which may include verbal persuasion from significant others.

**Table 2:** Two examples of the CMOCs

**CMOC’s**	**Plausible mechanisms: ‘what’**	**Contexts: ‘for whom’ and ‘in what circumstances’**	**Possible outcomes**	**Measures**
CMOC 1	M1: Receiving feedback from the system might improve the user’s confidence by confirming performance.	C1: A system that is accessible (in the home setting) and used by the stroke survivor, independently of the therapist.	O1: Adoption and development of a self-management approach to rehabilitation (behaviour change).	Observation of use and avatar replays.
			- Independent rehabilitation, self-evaluation and self-monitoring of recovery.	User diary.
				Usage of the system.
				Interview data.
CMOC 2	M2: By receiving feedback, users might feel confident to be able to interpret their performance and changing their movements to improve subsequent performance(s).	C2: A system that can be used independently by the stroke survivor in the home.	O2: Development of self-management skills.	Observation of use and avatar replays.
			- Problem-solving.	Interview data.

### CMOC’s

The CMOCs are set out as: *Context (C) + Mechanism (M) = Outcomes (O)* - ‘what’ (M) works ‘for whom’ and in ‘what circumstances’ (C) to produce what possible outcomes (O). How these outcomes are measured is also presented in Table 2.

The CMOC’s are then tested and refined which enables the researcher to evaluate *‘what works for whom and in what circumstances and respects?’*

### Validating and exploring existing and New CMOC’s

The next process within the RE cycle (Figure 3) involves testing and validating the embryonic hypotheses by gaining the perspectives of those who are deemed important stakeholders in the delivery of the intervention. This enables the researcher to gain an empirical insight and explore existing and new propositions. In other words, *‘this is my theory, what is yours?’*[28].

### CMOC refinement through stakeholder involvement

#### Professional perspectives of the delivery of feedback in clinical practice

Separate focus groups [32,33] with Occupational therapists (OT) and Physiotherapists (PT) (n = 14) that were specifically involved in facilitating physical rehabilitation to stroke survivors in the patients’ home were convened to:

• Explore the pre-existing context and mechanisms underpinning the delivery of feedback during community post-stroke rehabilitation to validate the embryonic hypotheses.

• Establish the empirical theory underpinning what current practice and provision of information and extrinsic feedback therapists employ to assist patients to continue with their rehabilitation post hospital discharge.

The following Tables 3 and 4 detail the demographics of the therapists.

**Table 3:** Community stroke team one demographics

**Therapist**	**OT/PT**	**Years qualified**	**Qualification**	**Stroke speciality (time working in stroke in years)**
**HPA**	OT	20	Dip Cot	10
**HPB**	PT	10	Dip Grad Phys	7
**HPC**	PT	6	BSc (Hons)	2
**HPD**	PT	6	BSc (Hons)	1
**HPE**	PT	5	BSc (Hons)	1
**HPF**	OT	11	BSc (Hons); MSc OT	8
**HPG**	PT	8	BSc (Hons); MSc Mod asic Bobath	6
**HPH**	OT	6	BSc (Hons); Previous BSc (Hons)	2
**HPI**	OT	1	BSc (Hons)	1

**Table 4:** Community stroke team two demographics

**Therapist**	**OT/PT**	**Years qualified**	**Qualification**	**Stroke speciality (time working in stroke in years)**
**HP1**	PT	6	BSc (Hons); Basic Bobath	2
**HP2**	OT	20	Dip Cot; MSc Mods Basic Bobath	15
**HP3**	OT	2	BSc (Hons)	1
**HP4**	PT	13	BSc (Hons); Basic Bobath; Adv Bobath; MSc Mods	3
**HP5**	OT	18	Dip Cot	14

The focus groups highlighted how therapists control the rehabilitation process; what they include and who they include. For example, the therapists appear to make clinical decisions based on their empirical knowledge and understanding, their evaluation of contextual factors and what they believe will be effective for that individual patient. It was also suggested that the provision of feedback must be adaptable and personalised towards the specific personal and environmental context of the recipient. Therefore, in order for the mechanisms to work, the context must include a system that is adaptable and can be personalised for the user.

#### Preliminary user testing

Following the acquisition of all necessary institutional ethical and governance approvals, two participants who had experience of testing the system and their co-resident carers were recruited to the study for the purposes of further refining the CMOC’s. They agreed to have the technology installed in their house and were interviewed before installation and after it had been in their home for around three days. Semi-structured interviews [34,35] were audio-taped to ensure the transcriptions were presented verbatim. In addition, observations [36,37] and field notes were taken to account for informal discussion and physical behaviour.

This initial testing revealed that firstly, a stroke survivor may adopt a self-managed approach to rehabilitation if they do not believe that they may become more socially isolated as a result of carrying out rehabilitation independently (i.e. receiving less help from others); and secondly, their motivation to set goals towards recovery is subject to having a continued desire to recover [19].

The interview data also suggested that the carers were able to engage in the rehabilitation process because they did not feel that when they provided feedback to the stroke survivor, they were being critical of the person they care for [19]. This was because the SMART system provides feedback instead of the carer. This allowed for a new CMOC hypothesis to be generated and subsequently tested in that ‘providing feedback through technology will enable significant others to take a more active role in the rehabilitation process and reinforce behaviour.’

### Systematic testing of the CMOC’s

The multi-method collective case-study approach using multiple qualitative and quantitative observations enables exploration of how the same mechanisms play out in different contexts, producing different outcomes [27,38,39].

### Sampling

For both the preliminary and systematic testing, purposive sampling was used to identify participants who had recently been discharged from a period of multidisciplinary community stroke rehabilitation and also met the inclusion/exclusion criteria. The researcher was assisted in the identification of potential participants by the members of the Community Stroke Teams. All five participants approached for the study by the therapist(s) were recruited. Table 5 describes the participant demographics (pseudonyms are used).

**Table 5:** Demographics of the participants (pseudonyms are used)

**Case study**	**Age**	**CVA/Side affected**	**Time since stroke**	**MMSE score/30**	**FAST score/30**	**Computer experience**	**Active range of movement (affected shoulder)**
**Mr Brown**	70	L/R Hemi	6 months	25	28	+ (minimal)	90° Flexion
							45° Abd
**Mrs Green**	79	L/R Hemi	8 months	23	25	- (none)	70° Flexion
							70° Abd
**Mr Gray**	62	R/L Hemi	5 months	30	30	++ (moderate)	30° Flexion
							20° Abd
**Mr Blackwell**	65	L/R Hemi	5 months	30	29	+++ (extensive)	20° Flexion
							20° Abd
**Mr Redmond**	79	L/R Hemi	5 months	27	25	+ (minimal)	90° Flexion
							90° Abd

### Selection criteria

• Inclusion:

○ A definite diagnosis of stroke (as reported by the therapist(s) through medical records) and have not been referred for further rehabilitation and/or receiving further rehabilitation.

○ Able to give informed consent to participation in the study and for their G.P. to be informed of their involvement

○ Living with co-resident carer

• Exclusion:

○ Unable to speak or comprehend written English.

○ Presence of aphasia (Frenchay Aphasia Screening Test (FAST) score of ≤ 24) [40].

○ Severe communication, perceptual or cognitive disorders (Mini Mental State Examination (MMSE) score of ≤ 17) [41].

○ Visual impairment.

○ Apraxic.

○ Shoulder subluxation or arm pain.

○ Medically unstable and other neurological, neuromuscular, or orthopaedic disorders that would interfere with task performance.

The following table describes the participants involved in this study.

### Research setting

Following screening of the volunteers with stroke using FAST and MMSE informed consent was obtained firstly from the person with stroke and then from their co-resident carer. All researcher contact with participants and their carers was in the participants’ home. These allowed for interviews and observations to be conducted in a natural environment as well as meet the aims of the study; home based rehabilitation using technology.

### Procedures

Following acquisition of all necessary National Research Ethics approval from the South Yorkshire Research Ethics Committee (Ref: 08/H1310/63) and governance approval from the Sheffield Health and Social Research Consortium, the SMART system was provided for individual participants to use for up to five weeks in their homes (from initial face-to-face contact to removal of the system). Following consent, participants and carers were both instructed in how to use the system and were also provided with a hard copy user manual. The people with stroke was then invited to undertake upper-limb rehabilitation using the SMART system as frequently as they wished during the five weeks and were visited for data collection and system support purposes.Each participant was initially advised to undertake reach forward and reach sideways exercises. This was because they were simple exercises that enabled the user to see the avatar move in different planes. Depending on observed and reported ability (and limitations) to perform the exercises and the participant’s expressed desire to try more exercises, they were then given others to attempt such as, hand-to-mouth; and a ‘catch the ball’ game. During and following prescribed activity; computer feedback was provided via real-time 3-D images; a qualitative chart and a graph (Figure 2a, d) displayed on a lap-top computer.

The participants were not advised as to the frequency of usage or the number of repetitions to perform but were encouraged to use the equipment as often as they wished which were then logged by the SMART system. This allowed the researcher to evaluate the number of sessions the participant chose to do. This provided some insight of the participant’s willingness to use the equipment. However, they may have felt obliged to use the equipment by agreeing to participate in the study.

### Data collection and methods used

**Figure 5:**
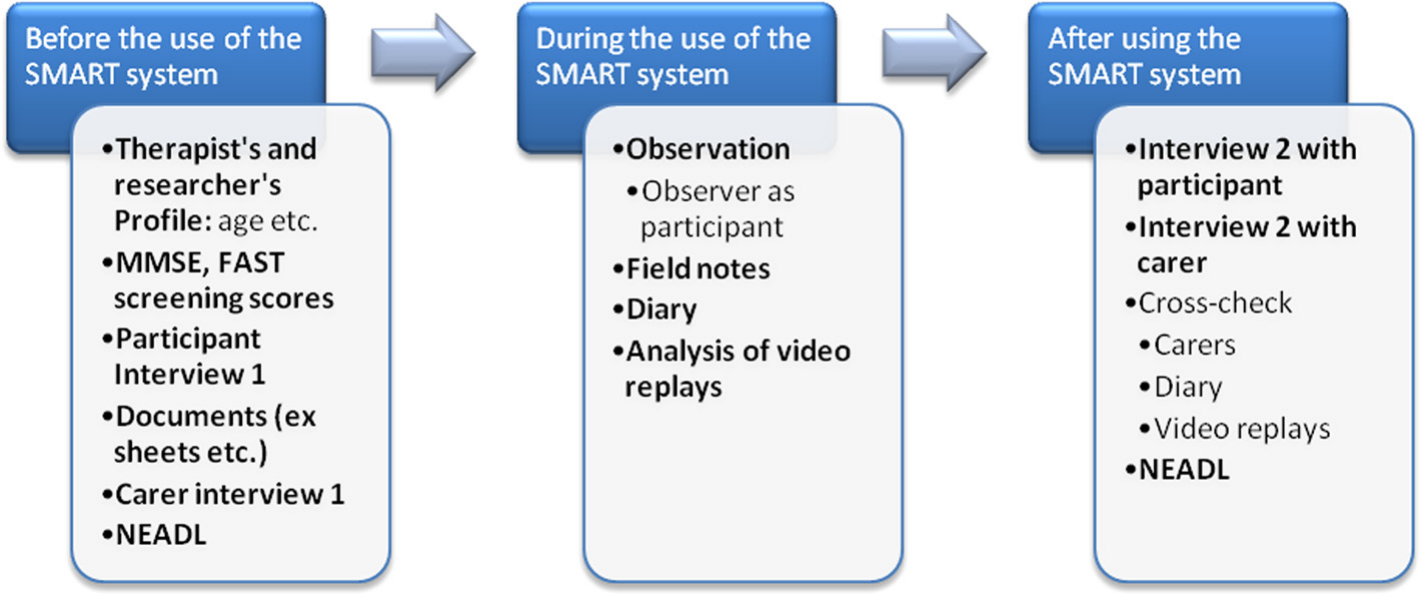
Data collection methods before, during and after using the SMART system.

The case studies involved a number of methods of data collection before during and after technology use to explore the context of the participants; observe the mechanisms involved in using the technology; and explore the outcomes (Figure 5) [32-37,42-44].

### Data analysis

Data analysis was based on both the exploration of the pre-existing context of the participant and the development and refinement of the hypothesised CMOC’s using thematic and framework analysis [45,46]. This innovative approach to the analysis draws on Yin [39], Miles and Huberman [46] and Patton [47] and is underpinned by the principles of Pawson and Tilley’s RE [27]. This approach allowed for themes to emerge from multiple sources of data and examines interconnections and relationships between the mechanism(s) and context(s) in relation to proposed outcomes [48,49].

## Results

The next stage of the RE cycle (Figure 3) involves the specification phase where the findings are synthesised and presented as refined CMOC configurations to answer the question: ‘what works for whom and in what circumstances and respects?’ [27,28].

### ‘What work works for whom and in what circumstances and respects’

Data analysis reveals that in order to achieve desired outcomes through the use of computer technology, key elements of computer-feedback such as; accurate, measurable, rewarding, adaptable, and knowledge of results feedback are required to trigger the mechanisms underpinning the intervention. In addition, the pre-existing context and the personal and environmental contexts such as; previous experiences of service delivery, personal goals, trust in technology, social circumstances and practicalities may also enable or constrain the underpinning theory-driven mechanisms.

Throughout the observational phase, a number of specific theoretical components were found to be of particular importance including; receiving feedback that was perceived as being rewarding, accurate, and included the results of performance (knowledge of results feedback) that was measurable. These components enabled key mechanisms to work such as motivating the users (and carers) to use the equipment and having the confidence to continue using it. This also impacted on their engagement with the system and frequency of usage which in turn served to reinforce self-managed rehabilitation.

### Adaptable and personalised feedback

In order to improve the users’ ability to relate to the avatar, it was suggested that the graphical interface needs to be individualised to the user. For example, the user may wish to alter the avatar image to look like them (i.e. male/female) and therefore, make it both recognisable and easier to relate to. One user found it difficult to relate to the avatar as she thought it did not represent her,

“It’s not me on the screen, that’s a man and I’m a woman!” (Mrs Green)

In addition, one participant explained that the feedback would be more meaningful if his results were something he could relate to in everyday life like being able to play his guitar or using his hand to hold a plectrum,

“The feedback would be better if I could relate it to playing my guitar or holding the plectrum because these are things I want to be able to do… goals” Mr. Blackwell.

This suggests that further developments in feedback should be capable of being tailored towards the user’s specific goals to enable the feedback to become ‘meaningful’ to the user.

### Rewarding feedback

The provision of rewarding feedback impacted on the participant’s motivation to use the system. Three people with stroke specifically described how obtaining positive results affected their motivation to use the system. This was confirmed by Mrs. Green’s carer who suggested that once she had managed to achieve good results, she was determined to maintain her performance which resulted in her wanting to use the system more often (increased usage).

“It was only when she got all the dots in the green she really wanted to go on it again” (Mrs Green’s carer).

### Accuracy and reliability

Whilst positive, rewarding feedback motivated the users this was offset by a lack of overall reliability of the technology. For example, one of the users described that if he observed differences in the on-screen feedback compared with how he perceived he had performed, he would lose trust in the technology,

“I was expecting better results than that… I thought I had done well but that [the computer] says I haven’t… It’s disappointing; it must be the sensors” Mr Gray.

### Knowledge of results and measurable feedback

All of the users were uninterested in watching their replays using the avatar (knowledge of performance feedback). One participant explained how the qualitative knowledge of results chart was easier to understand and enabled her to interpret how well she had performed,

“I didn’t really grasp the two men on the screen. Those dots were much easier but it still took a few go’s to get used to it. I knew if I got in the green I‘d done well” Mrs. Green.

Explicit knowledge of results such as, the provision of scores with the ‘catch the ball’ game (Mr Redmond) also increased the determination of the user to improve,

“It’s a good idea; you can see a target to aim for! I can see the dots getting higher and my score going up on that game” Mr. Redmond.

Mr Redmond’s carer suggested that providing a score also enabled him to tell others about his progress, leading to him receiving praise from others, which reinforced his engagement with rehabilitation.

“It is easier for him to tell other people that ‘I did catch the ball and I got 3100’ or… ‘I’m better this week I got 3600’ and even though it might not make much sense to other people, they can tell it is going up and it always seems to appeal to him and he wants to do better on it each time” Mr Redmond’s carer.

### Contextual factors

Contextual factors also influenced the activation of mechanisms. These included; the pre-existing context, the personal and environmental context, and the influence of the carer and researcher. These are described below.

*Pre-existing context* - Previous experience of rehabilitation (i.e. therapist led rehabilitation to patient led rehabilitation) could influence expectations of service delivery. One participant did not believe it was his responsibility to lead his rehabilitation,

“It’s not up to me to sit here and do it all myself is it… You’re the experts you should be doing it” Mr Brown.

*Personal and Environmental context* - This included personal preferences, stroke severity, acceptance of technology and motivation to improve and carry out rehabilitation independently which the research confirmed all impacted on the utilisation of feedback from the technology. In addition, the environmental restrictions of space and ferromagnetic interference also detrimentally influenced the use of the SMART system. For example, not having somewhere to store the SMART system when not in use and the sensitivity of the inertial sensors to large metal objects (i.e. radiators) resulted in the SMART system being stowed away upstairs. In other words, if the sensors were kept near a large metal object, this would affect the accuracy of the sensors and distort the on-screen image. Because of this moving the system from its place of use to its place of storage increased the amount of setting up time and effort required prior to use.

*Carer and researcher influence* - For the carer, issues such as personal skills, feeling empowered to be involved, accepting technology in the home and as part of a therapeutic package were also key to the utilisation of the SMART system. For the researcher, the provision of adequate training and support was essential.

### Quantitative outcomes: the Nottingham Extended Activities of Daily Living (NEADL) scores

Analysis of the NEADL scores [42-44] for each participant revealed that irrespective of the number of attempts to use the SMART system or the number of sets and repetitions, the participants did not improve their measured functional independence over the period they had the technology in their home. This could be attributed to a number of factors including the insensitivity of the chosen outcome measure, the specificity of the computer exercises and the number of repetitions carried out. Furthermore, it should be noted that all of the participants were ≥ five months post-stroke and only used the system for up to five weeks. This may therefore not have been a long enough period of time to drive neuroplasticity and functional improvement [50].

### CMOC refinement

This research aimed to test and refine intervention theories by exploring the complex interactions of contexts, mechanisms and outcomes. This involves refining CMOC propositions which can then be retested (Figure 3).

The combination of determining what works/does not work for whom in what circumstance and respects has identified elements of feedback that are essential for mechanisms to work. This has enabled the refinement of theories underpinning the use of computer-based feedback for post-stroke upper-limb rehabilitation in the home. Indeed, this research has made significant refinements to the proposed CMOC’s. Two examples of these refinements are set out in Table 6. The full table of refined CMOC’s are available as an Additional file 1.

**Table 6:** The Refined CMOC’s

**CMOC’s**	**Plausible mechanisms: ‘what’**	**Contexts: ‘for whom’ and ‘in what circumstances’**	**Possible outcomes**
CMOC 1	M1: Receiving **rewarding, KR** feedback from the system might improve the user’s confidence by confirming performance.	C1: A system that:	O1: Adoption and development of a self-management approach to rehabilitation (behaviour change).
		• **Is reliable, accurate and robust.**	
		• **Can be adapted and personalised to the individual personal, environmental and social context of the stroke survivor**	- Independent rehabilitation, self-evaluation and self-monitoring of recovery.
		• Is accessible in the home setting	
		• Is used by the stroke survivor **who does not believe they will become more socially isolated as a result of carrying out rehabilitation and subsequent ADL’s,**	
		• Can be used independently of the therapist.	
CMOC 2	M2: By receiving **KR and KP** feedback, users might feel confident to be able to interpret their performance and changing their movements to improve subsequent performance(s).	C2: A system that:	O2: Development of self-management skills.
		• **Is reliable, accurate and**	- Problem-solving.
		• **robust.**	
		• **Can be adapted and personalised to the individual personal, environmental and social context of the stroke survivor**	
		• Can be used independently by the stroke survivor in the home.	
		• **Is provided in an environment where the user is provided with adequate resources and support.**	

## Discussion

Existing literature suggests that concurrent feedback, knowledge of performance (KP), knowledge of results (KR) and explicit feedback may be key components in the promotion of improved performance. However, existing literature also highlighted the heterogeneity in studies that have explored the use of feedback in post-stroke rehabilitation [51]. This suggests that careful consideration should be made as to what form and method of delivery of feedback is given. In other words, the provision of feedback should not be a ‘one size fits all’ component of rehabilitation and different forms and methods of delivering feedback may be more effective at promoting self-managed rehabilitation in different contexts [51-53].

The findings have revealed that the mechanisms underpinning the utilisation of feedback from computer technology for upper-limb rehabilitation in the home are influenced by the pre-existing, personal and environmental contextual factors surrounding the user. The identification of the interaction between feedback, mechanisms and outcome(s) has also revealed how underpinning theories (theoretical components) may work in combination with other theories to produce an outcome within a given context. For example, knowledge of results (feedback theory) [21] provided confirmation of performance (self-efficacy) [54] which motivated the user (Social Cognition Theory) [55], this gave the user the confidence (self-efficacy) [54] to use the system. This led to increased usage, repetitive use (motor-learning) [29,31] and increased their familiarity and use of the system (resource utilisation) [56]. In turn this repetitive use probably facilitated motor learning and improved results (motor learning) [29,31], reinforcement and increased motivation (Social Cognition Theory) [55]. This has highlighted the key ingredients that are necessary in activating the mechanisms underpinning the utilisation of computer-based feedback as well as the issues involved in testing early prototype technology and how developing technology systems need to be reliable, robust and accurate.

This research has highlighted the need for computer feedback to be both accurate and reliable. Indeed, the accuracy (i.e. it would show the arm moving backwards when it is moving forwards) and reliability (i.e. the system would crash and/or not boot up correctly) of the equipment used in this study influenced the utilisation of feedback which in turn, hindered the mechanisms. Because the on-screen display was sometimes inaccurate, the participants (and in some instances the carers as well) became frustrated, lost patience, and trust in the feedback provided. This resulted in the participants being less willing to use the equipment and/or dismissing the feedback as an inaccurate evaluation of their performance. Therefore, if similar systems are to be used in the future for mainstream rehabilitation, accuracy and reliability is essential.

However, despite the encouraging qualitative findings for the promotion of self-managed rehabilitation, the quantitative findings suggest that this did not result in functional improvement. Therefore, further work is required to explore how overcoming technological and contextual challenges may lead to the increased intensity and longer periods of use (months not weeks) which will drive neuroplastic adaptation [49].

Developing technology needs to account for the clinical needs of the practitioners as well as the end users themselves. If technology is to be used in mainstream therapy, service providers are required to consider the impact and variance of the context, the reliability and accuracy of the technology and importantly, what forms of feedback are provided to facilitate the users’ understanding of performance.

To have clinical utility, technology systems would need to incorporate greater clinical adaptability, such as, including greater variety of exercises into the programme(s). For example, the PhysioTools (http://www.physiotools.com) [57] exercise sheets used currently (and by the therapists in this study) has over 16000 activities that can be selected and subsequently prescribed by the therapist. This enables therapists to choose from a vast library of activities what to prescribe making their therapy specific to the requirements of the patient. Therefore, if therapists are to incorporate technology into their practice, they may require and even expect the technology to be a better alternative to what is currently available to them.

With the growing interest in gaming technology such as the Nintendo® Wii™; future users may be more receptive to computer interaction; however, they may also have higher expectations in terms of the interface and motivational components of rehabilitative devices. Therefore, if future systems are to be embraced they will have to meet the rising expectations of system users and therapists for both usability and clinical utility.

Research also suggests that therapists currently provide a model of service delivery that is led by their empirical knowledge, which may not be conducive to motor learning and self-management. This may therefore limit the stroke survivor’s ability to utilise computer-based feedback to facilitate self-managed rehabilitation. Work is required to educate therapists in the provision of therapy that includes newer innovative methods of delivering feedback that facilitates independent rehabilitation. This will place less demand on services whilst empowering stroke survivors and close family members to carry out and continue their recovery beyond the acute and sub-acute period.

Finally, the adoption of a realistic evaluation methodological approach used in this research gave an important innovative dimension to the study since this methodology has not been used in the development of technology for rehabilitation. In line with the MRC framework for complex interventions [58], this approach has allowed for the refinement of the theories underpinning the intervention within the context of delivery and indeed tested the adoption of realistic evaluation for on-going research [23].Further research is required to continue the cycle set out in Figure 1 where the refined CMOC’s should be tested again with the refinements in place. This would involve changes to service delivery, therapy education, and the technology itself (as described previously). This would allow for further testing of the theoretical underpinnings of this intervention and the exclusion of the problematic non-conducive elements highlighted in this study.

The SMART 2 project is currently exploring the implementation and adoption of the key elements of feedback highlighted in this research through the development and user testing of the Personalised Self-Management Rehabilitation System for stroke (SMART 2) [23].

## Conclusions

This research has exposed the interaction between the feedback delivered through computer technology, the mechanisms underpinning the intervention, the context, and how this leads to variable outcome(s).

The identification of these interactions may therefore inform the development of technology; therapy education and the subsequent adoption of technology and a self-management paradigm; long-term self-managed rehabilitation; and importantly, improvements in the physical and psychosocial aspects of recovery. Further work is required to; develop technology so that it incorporates the elements of feedback highlighted by this research; ensure the technology is robust, reliable and accurate; investigate the clinical utility of technology for home-based stroke rehabilitation, and the extent to which it might encourage utilisation by the end user.

## Abbreviations

ICT: Information and communications technology; EPSRC: Engineering and Physical Sciences Research Council; MR: Medical Research Council; RE: Realistic evaluation; SMART: Self-Management supported by Assistive, Rehabilitation and Telecare Technologies; FAST: Frenchay aphasia screening test; MMSE: Mini Mental State Examination; NEADL: The Nottingham Extended Activities of Daily Living Scale; CMOC: Context-mechanism-outcome configuration.

## Competing interests

The authors declare that they have no competing interests.

## Authors’ contributions

JP carried out the research, participated in its design and coordinated the manuscript. SM assisted in the study design and coordination and helped to draft the manuscript. GM assisted in the study design and helped to draft the manuscript. NN helped to conceptualise the methodological application and draft the manuscript. HZ provided the technical expertise and helped to draft the manuscript. All authors read and approved the final manuscript.

## Pre-publication history

The pre-publication history for this paper can be accessed here:

http://www.biomedcentral.com/1472-6947/14/46/prepub

## Supplementary Material

Additional file 1 Additional Material: Refined CMOCs. Click here for file
