# Supplementary material for: Stroke patients’ utilisation of extrinsic feedback from computer-based technology in the home: a multiple case study realistic evaluation
Source: BMC Med Inform Decis Mak. 2014 Jun 5;14:46. doi: 10.1186/1472-6947-14-46 (PMC4070341; doi:10.1186/1472-6947-14-46)
Supplement: Additional file 1 — Additional Material: Refined CMOCs. [file 1472-6947-14-46-S1.docx]

**Additional Material: Refined CMOCs**

| **The Refined CMOC’s** | | | |
| --- | --- | --- | --- |
| **CMOC’s** | **Plausible mechanisms: ‘what’** | **Contexts: ‘for whom’ and ‘in what circumstances’** | **Possible outcomes** |
| CMOC 1 | M1: Receiving rewarding, KR feedback from the system might improve the user’s confidence by confirming performance. | C1: A system that:   - Is reliable, accurate and robust. - Can be adapted and personalised to the individual personal, environmental and social context of the stroke survivor - Is accessible in the home setting - Is used by the stroke survivor who does not believe they will become more socially isolated as a result of carrying out rehabilitation and subsequent ADL’s, - Can be used independently of the therapist. | O1: Adoption and development of a self-management approach to rehabilitation (behaviour change).   - Independent rehabilitation, self-evaluation and self-monitoring of recovery. |
| CMOC 2 | M2: By receiving KR and KP feedback, users might feel confident to be able to interpret their performance and changing their movements to improve subsequent performance(s). | C2: A system that:   - Is reliable, accurate and robust. - Can be adapted and personalised to the individual personal, environmental and social context of the stroke survivor - Can be used independently by the stroke survivor in the home. - Is provided in an environment where the user is provided with adequate resources and support. | O2: Development of self-management skills.   - Problem-solving. |
| CMOC 3 | M3: By receiving rewarding KR feedback, users might feel confident in making decisions, utilising resources, and taking action. | C3: A system that:   - Is reliable, accurate and robust. - Can be adapted and personalised to the individual personal, environmental and social context of the stroke survivor - Provides feedback to the stroke survivor during and after use. - Is provided in an environment where the user is provided with adequate resources and support. | O3: Users take ownership of their condition.   - Making decisions, utilising resources, and taking action. |
| CMOC 4 | M4: By receiving measurable, rewarding KR feedback, users might engage with significant others such as, the therapist and carer. | C4: A system that:  A system that:   - Is reliable, accurate and robust. - Can be adapted and personalised to the individual personal, environmental and social context of the stroke survivor - Involves significant others. | O4: Users involve others in their rehabilitation through choice.   - Collaborate with significant others. |
| CMOC 5 | M5: By receiving meaningful feedback, users might be motivate to set specific, measurable, realistic, and time specified goals (targets) to achieve that are confirmed by the computer feedback. | C5: A system that:   - Is reliable, accurate and robust. - Can be adapted and personalised to the individual personal, environmental and social context of the stroke survivor - Allows stroke survivors who continue to have a desire to continue the recovery process to set goals in relation to the feedback provided in collaboration with a therapist. | O5: Increased motivation and self-management.   - Goal-setting. |
| CMOC 6 | M6: By receiving feedback, users might feel confident to recognise inaccurate feedback and problem-solve by overcoming technical problems encountered that impact on the feedback provided. | C6: An environment where:   - The user is provided with adequate resources, training and support - The performance of the equipment is not adversely affected. - The equipment is easily accessible for the user. | O6: Development of self-efficacy.   - Perseverance in adversity. |
| CMOC 7 | M7: By receiving measurable, rewarding feedback, users might be motivated to use the SMART system as a result of positive reinforcement. | C7: A system that provides feedback that can be described and/or observed by significant others. | O7: Increased usage of the affected upper-limb, intensity, and repetition. |
| CMOC 8 | M8: By receiving rewarding feedback, users might be motivated to carry out intense, repetitive, specific, salient practice. | C8: A system that:   - Is reliable, accurate and robust - Can be adapted and personalised to the individual personal, environmental and social context of the stroke survivor - Is provided in an environment where the user is provided with adequate resources and support. | O8: Improved motor and functional performance. |
| CMOC 9 | M9: By receiving rewarding feedback, users might be more confident and motivated to carry out repetitive practice and increase their usage of their affected upper-limb. | C9: A system that encourages the stroke survivor to carry out computer exercises repetitively. | O9: Increased use of the affected upper-limb in ADL’s and social participation. |
| CMOC 10 | M10: The delivery of rewarding feedback might engage the users. | C10: A system that:   - Is reliable, accurate and robust. - Can be adapted and personalised to the individual personal, environmental and social context of the stroke survivor - Is provided in an environment where the user is provided with adequate resources and support. | O10: Increased repetition through the use of the SMART system. |
| CMOC 11 | M11: The delivery of concurrent feedback might engage the users and enable them to learn implicitly through trial and error. | C11: A system that:   - Is reliable, accurate and robust. - can be adapted and personalised to the individual personal, environmental and social context of the stroke survivor - Is provided in an environment where the user is provided with adequate resources and support. | O11: Improved performance on the computer exercises. |
| CMOC 12 | M12: Providing measurable, rewarding KR feedback through technology may enable significant others to take a more active role in the rehabilitation process and reinforce behaviour. | C12: A system that:   - Is reliable, accurate and robust. - Can be adapted and personalised to the individual personal, environmental and social context of the stroke survivor - Allows stroke survivors to utilise the feedback in the presence of significant others. | O12: Significant others are involved in their rehabilitation through choice. |
